# The epidemiology of neurocognitive disorders in Hungary

**DOI:** 10.1038/s41598-026-44201-4

**Published:** 2026-03-17

**Authors:** Christopher Váraljai, András Attila Horváth, Anita Kamondi

**Affiliations:** 1 Department of Neurosurgery and Neurointervention, Semmelweis University, Laky Adolf utca 44, Budapest, 1145 Hungary; 2 Neurocognitive Research Centre, Nyírő Gyula National Institute of Psychiatry and Addictology, Lehel utca 59, Budapest, 1135 Hungary; 3 Doctoral School of Semmelweis University, Üllői út 26, Budapest, 1085 Hungary; 4 Department of Anatomy, Histology and Embryology, Semmelweis University, Tűzoltó utca 58, Budapest, 1094 Hungary; 5 Department of Neurology, Semmelweis University, Balassa u. 6, Budapest, 1083 Hungary

**Keywords:** Epidemiology, Neurocognitive disorder, Dementia, Hungary, Prevalence, Comorbidity, Health care, Neurology, Risk factors

## Abstract

There is a lack of data on the epidemiological characteristics of NCD (neurocognitive. disorder) in Hungary. We aimed to assess the prevalence and incidence of NCD in Hungary, by sex and age groups, and to determine the proportion of diagnostic procedures performed on patients, the prevalence of comorbidities within this population, and the medication used to treat these patients. Descriptive statistical analysis based on data from the National Health Insurance Fund and the Central Statistical Office. Data are for the years 2016 to 2021. The prevalence of NCD in people over 65 in 2016 was 6.64%, decreasing to 5.58% in 2021. The annual incidence in 2016 was 65,505, which decreased to 42,668 in 2021. 40% of incident. patients received a CT scan of the skull. Around 80% of patients were diagnosed with hypertension. 75% had cerebrovascular disease in 2016, which decreased to 59.3% in 2021. A third of patients were diagnosed with type 2 diabetes. In 2016, 8.99% of patients filled their prescriptions for NCD, this figure did not change significantly by 2021. The prevalence of NCD in Hungary decreased significantly after 2019, presumably due to the decreased use of healthcare facilities during the COVID-19 pandemic.

**Supplementary Information:**

The online version contains supplementary material available at 10.1038/s41598-026-44201-4.

## Introduction

Neurocognitive disorder (NCD) is a group of diseases characterised by a progressive cognitive decline. According to DSM-5 NCDs^1^ neurocognitive disorders are grouped into two main categories based on severity: (1) major neurocognitive disorders (formerly known as dementia) are conditions in which the patient’s cognitive performance declines to a degree that significantly impairs their ability to carry out everyday activities; and (2) mild neurocognitive disorders where patients’ cognition is affected but does not hinder their daily activities. The affected cognitive domains include complex attention, executive function, learning and memory, and language. There are multiple aetiologies ranging from various proteinopathies (most commonly amyloid, tau, and alpha synuclein) to loss of function caused by cerebrovascular diseases.

NCD carries a great burden for the affected patients, their relatives, and society. It causes serious psychological and social struggles, and it brings about significant direct and indirect financial costs^2^.

According to the estimations of Alzheimer Europe, 1.73% of the population of the European Union lives with major NCD^3^. However, it remains unclear how this number varies across countries in Eastern Europe, where epidemiological data on NCDs are limited^4^. In Hungary, the currently used figures are based on estimations derived from European data. However, due to various factors, for instance, differences in the overall health conditions of the Hungarian population, these estimations might not be appropriate. Our study aimed to determine the true prevalence and yearly incidence of NCD in Hungary between 2016 and 2021 and to specify its distribution in different age groups and among men and women. Furthermore, we obtained data on the number of diagnostic procedures used to establish the NCD diagnosis and also on the number of individuals who received prescriptions for anti-dementia medication. Certain comorbidities are associated with a higher mortality rate, worse quality of life, and greater use of health care services^5^ and some comorbidities can accelerate the progression of NCDs^6^. For these reasons we also examined the prevalence of comorbidities within the study population.

## Methods

### Data collection

Data were obtained from the Hungarian National Insurance Fund (NEAK). This dataset includes all Hungarians, who are covered by the national health insurance, encompassing about 95% of the population^7^. Whenever a diagnosis is made in the national healthcare service it must be coded as an ICD-10 code by a practitioner and uploaded to a central database. After this, the data reaches the national health insurance fund and health institutions are paid according to the Diagnosis-Related Groups (DRGs) system. We requested the anonymized data from NEAK and received the datasheets encompassing the number of patients diagnosed with NCD by gender, age-group, and year in the period of 2016–2021. If a patient had multiple diagnoses of NCD it counted as one case. If the diagnosis was made before 2016 the patient still appeared as a prevalent case in the data. Furthermore, we obtained a datasheet containing the number of diagnostic procedures performed on incident patients by year and how many patients filled a prescription of anti-dementia medication in the year of diagnosis.

All methods were carried out in accordance with relevant guidelines and regulations. The study was approved by the Health Science Council, Scientific and Research Ethics Committee (Egészségügyi Tudományos Tanács Tudományos és Kutatásetikai Bizottság, ETT TUKEB), Budapest, Hungary (ETT TUKEB BM/14265-3/2025). We confirm that the database used in this study contained fully anonymized data. No direct or indirect identifiers were available to the researchers, and individual patients could not be identified in any way. According to applicable national regulations, the use of fully anonymized retrospective data does not require informed consent from participants. We confirm that the study was conducted in accordance with relevant ethical guidelines and regulations for the use of anonymized health data.

### Prevalence and incidence data

Prevalent cases were defined as having a diagnosis of any type of NCD defined by the International Classification of Diseases 10th version (ICD-10) codes. All included ICD-10 codes can be found in Supplementary Table 1. We did not obtain data regarding the number of patients diagnosed with various NCD subtypes, only the overall number of people diagnosed with NCD.

Data included prevalence and incidence rates for the given year, both for men and women, comorbidities of patients diagnosed in the given year, diagnostic procedures used in the year of diagnosis or the preceding year, number of patients who filled their prescribed anti-dementia medication in the year following the diagnosis.

Population data were collected from the database of the Hungarian Central Statistical Office (KSH) for each examined year^8^.

### Comorbidity data

Comorbidity encompasses the combined effects of additional conditions in relation to the index condition in an individual^9^. We examined the coexistence of diseases that could contribute to the progression of cognitive decline in incident NCD patients, for instance, hypertension, cerebrovascular diseases, epilepsy, etc. The ICD-10 codes of all examined comorbidities are listed in Supplementary Table 1.

### Diagnostic procedures

We examined the proportion of patients who had relevant diagnostic procedures to validate their diagnosis. The included diagnostic procedures are used for the diagnosis of NCD according to the Hungarian dementia guideline, which was in effect at the time of the diagnosis^10^. Diagnostic procedures were collected based on the Hungarian version of the International Classification of Health Interventions (OENO). We collected the number of these procedures in incident NCD cases in the year of and in the year preceding the diagnosis. The following procedures were collected: serum levels of thyroid stimulating hormone (TSH), Vitamin B12, folate, and copper; the rapid plasma regain test for diagnosis of Treponema infection; investigation of cerebrospinal fluid; MRI or CT scan of the head; electroencephalography (EEG); and 6 types of neuropsychological investigations (NPT): (1) mini-mental state exam (MMSE), (2) psychiatric cross-sectional diagnosis, during which a psychiatrist or clinical psychologist forms an opinion on the psychological status of the patient, (3) mapping of cognitive functions, (4) examination of dementia (including at least one of the following tests: Blessed Dementia Score, Reisberg Functional Assessment of Dementia State, Ranschburg-Ziehen test, Hamilton, or Brief Psychiatric Rating Scale), (5) problem-centred neuropsychological testing, and (6) general neuropsychological examination.

### Data regarding the treatment of NCD

In Hungary, medications approved for the treatment of Alzheimer’s disease (AD) are donepezil and rivastigmine (acetylcholinesterase inhibitors) as well as memantine (NMDA receptor antagonist). Two other drugs, vinpocetin and piracetam, are also commonly prescribed for patients with NCD, however, their efficacy is highly disputed^11,12^. We collected information on the number of patients filling at least one prescription in the year or in the following year of their NCD diagnosis to reveal what proportion of the NCD population receives any of the above medications for their condition.

### Data analysis

Patients were divided into 6 age-groups: under 65 years of age, 65–69, 70–74, 75–79, and 80 years and older. We also created a common age group for all patients 65 years and over. We calculated the prevalence (%) and incidence (/1000 people/year) of NCD in the male and female population and in the various age groups. The crude rate of relevant diagnostic procedures in incident NCD cases and the use of medication were also calculated. These descriptive statistics were performed using Microsoft Excel 2016.

The trends in dementia prevalence and incidence rates for every age group and both sexes were calculated from 2016 to 2021, as well as separately for the period between 2019 and 2021, to assess the impact of the COVID-19 pandemic, using joinpoint regression analysis of the SEER*Stat software. The annual percentage change (APC) was calculated from this model. The trend of prevalent comorbidities in incident NCD cases and the trend of anti-dementia prescriptions filled in the year of diagnosis were also calculated. The correlation between age groups and the number of prevalent cases of NCD was calculated using JASP 0.18.3.0 with Pearson’s correlation.

## Results

### Study population

Between 2016 and 2021, there were a total of 312,781 cases diagnosed with NCD in Hungary. More than half (*n* = 197,052; 63%) of the study population was women, and this ratio remained constant over the examined period. Among the incident cases, 87,012 were under the age of 65 (1.15:1 female to male ratio), 29,443 were 65–69 years of age (1.27:1 female to male ratio), 36,601 were 70–74 years of age (1.52:1 female to male ratio), 47,018 were 75–79 years of age (1.87:1 female to male ratio), and 112,359 were over the age of 80 (2.74:1 female to male ratio).

### Prevalence and incidence data

**Fig. 1:**
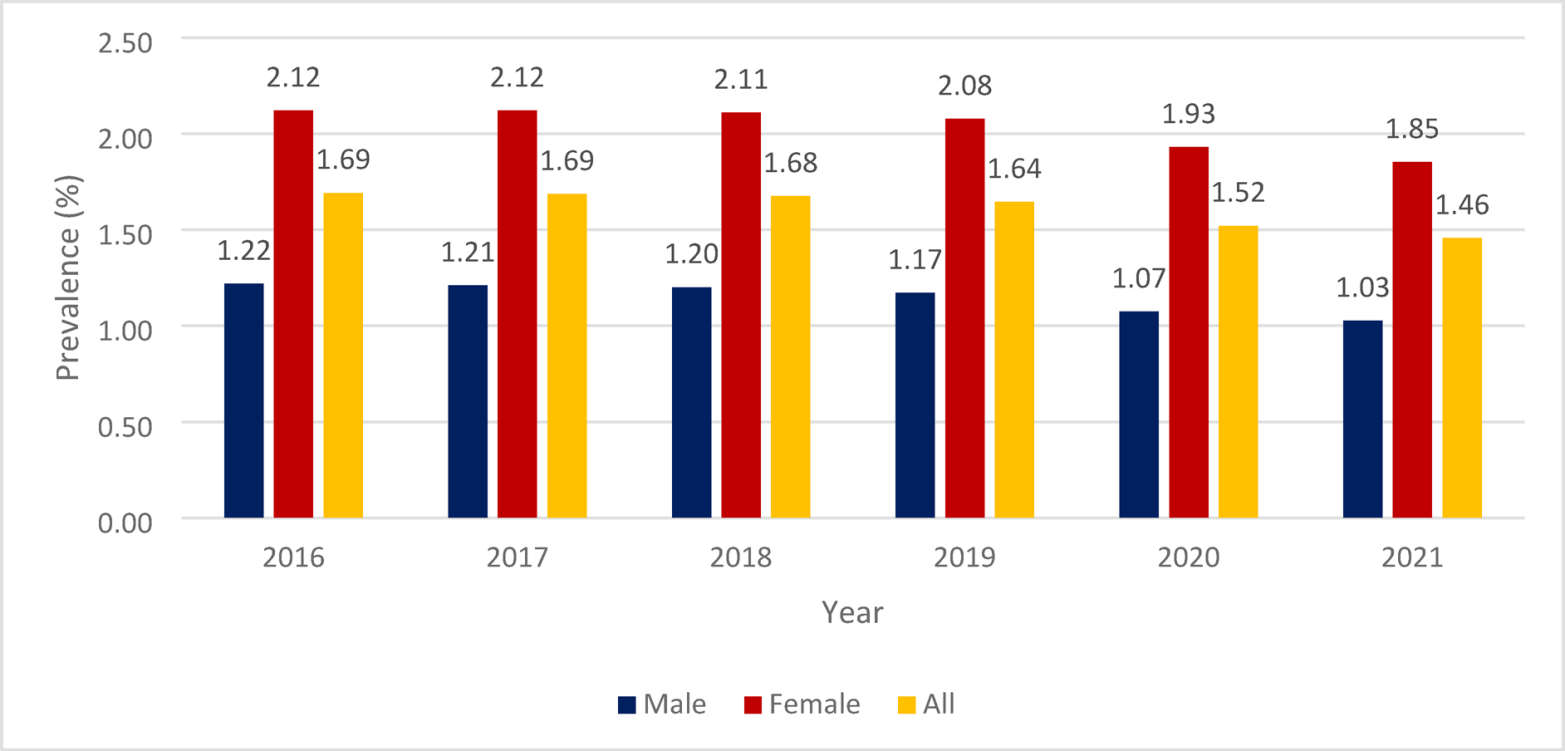
The prevalence (%) of NCD in the observational period (2016–2021).

The prevalence of NCD by year is shown in Fig. 1. For the whole population, the prevalence of diagnosed NCD cases decreased from 1.69% (*n* = 166,282) in 2016 to 1.46% (*n* = 141,744) in 2021. There was a significant negative trend between 2019 and 2021 (APC= −5.89; *p* < 0.001).

For the male population, the prevalent cases decreased from 1.22% in 2016 to 1.03% in 2021, and there was a significantly decreasing trend between 2019 and 2021 (APC=−6.42; *p* < 0.001). As for women, prevalent cases decreased from 2.12% in 2016 to 1.85% in 2021, and there was a significant negative trend between 2019 and 2021 (APC=−5.58; *p* < 0.001).

There was no significant trend observed for the whole study period (2016–2021) in either population.

**Fig. 2:**
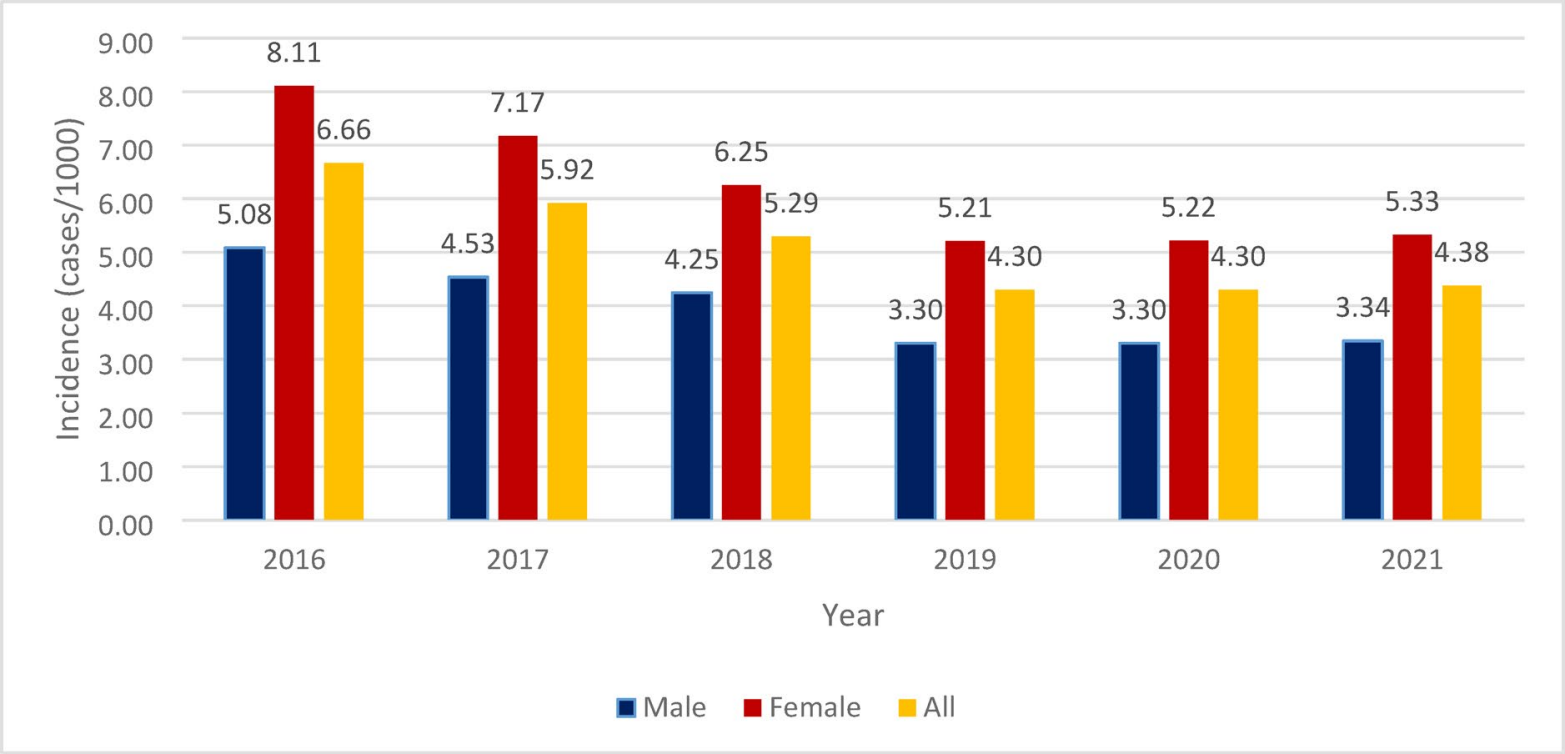
The incidence/1000 people with NCD diagnosis over the observational period (2016–2021).

The yearly incidence data (NCD diagnosis/1000 people in the given year) are displayed in Fig. 2. The average yearly incidence for the entire population throughout the study period was 5.14/1000/year. Yearly incident cases decreased for the entire population from 6.66/1000 (*n* = 65,515) in 2016 to 4.38/1000 (*n* = 42,590) in 2021, and a significant negative trend was observed (APC=−8.55; *p* < 0.001). There were significant negative trends observed in the male and female populations (APC − 8.53; *p* < 0.001 and APC − 8.52; *p* < 0.001, respectively). There were 1.7 times as many incident cases in women as in men. In contrast with data for prevalent cases, the negative trend observed for incident cases between 2019 and 2021 was not significant in either population.

**Fig. 3:**
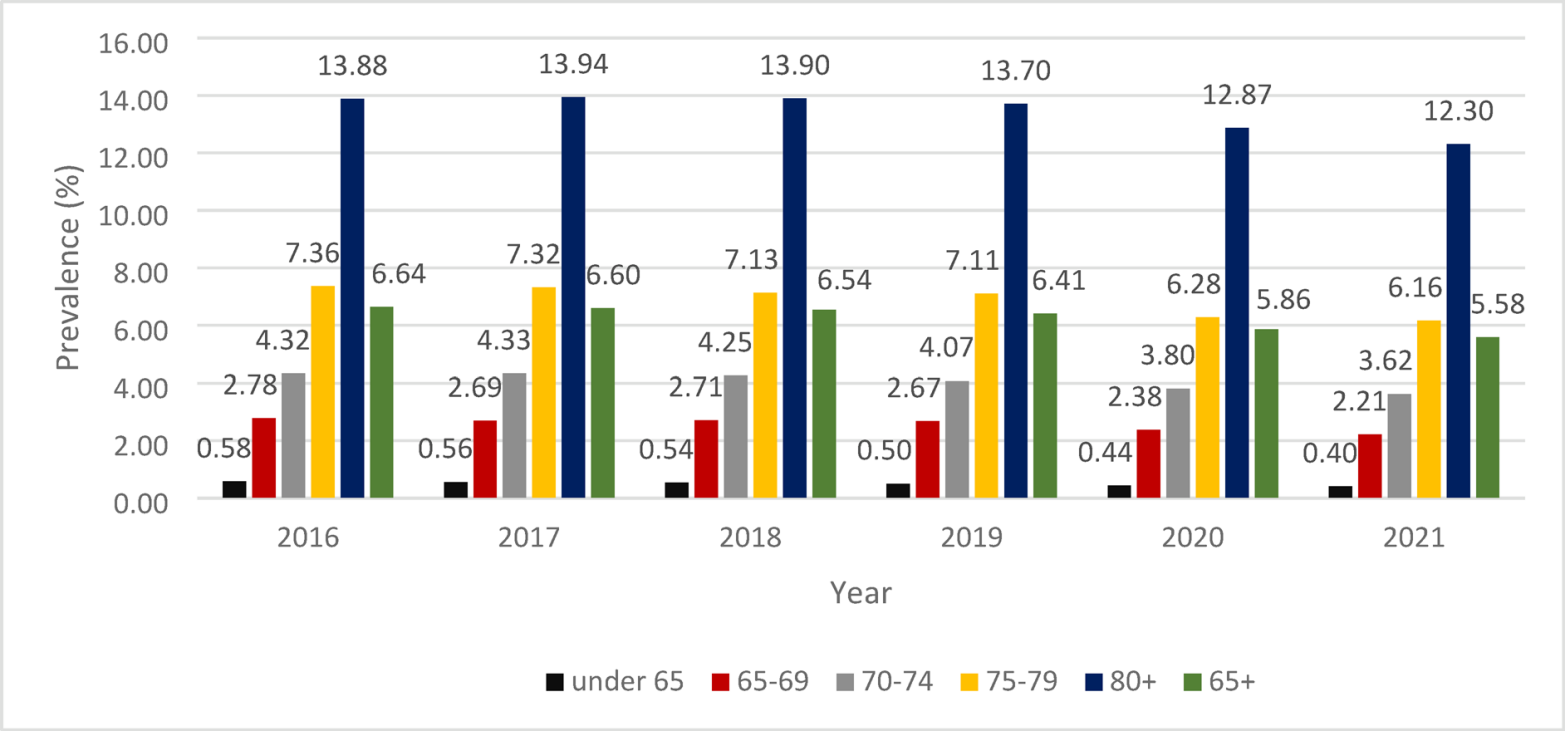
The prevalence (%) of NCD in the different age groups by year.

The prevalence data of NCD for specific age groups over the study period are presented in Fig. 3. As it is expected, the prevalence of NCD increases with age (Pearson’s *R* = 0.949; *p* < 0.001). In the age-groups of under 65, 65–69, 70–74 there was a significant negative trend between the years of 2016 and 2021 (under 65 - APC − 7.18; *p* < 0.001,65–69 – APC − 4.25; *p* = 0.041, 70–74 – APC − 3.7; *p* = 0.04). These populations also showed a significant negative trend between 2019 and 2021 (under 65 - APC=−10.24; *p* < 0.001; 65–69 – APC − 9.00; *p* < 0.001, 70–74 – APC − 5.72; *p* < 0.001). There was also a significant negative trend in this period for the 75–79 and over 80 age-groups (APC − 6.90; *p* < 0.001, APC − 5.24; *p* < 0.001, respectively).

The prevalence of NCD for people over 65 years of age decreased from 6.64% in 2016 to 5.58% in 2021. This population showed a significant negative trend over the whole study period (APC=−3.49; *p* = 0.013) and between 2019 and 2021 (APC=−6.66; *p* < 0.001). Regarding the absolute number of prevalent cases, the female to male ratio increased with age: for patients under 65 the ratio was in the range of 1.17–1.2 (depending on the year) and the over-80-year population the ratio was in the range of 3.18–3.27 (The exact number of prevalent cases for each age-group by sex can be found in Supplementary Table 2).

### Comorbidity

**Fig. 4:**
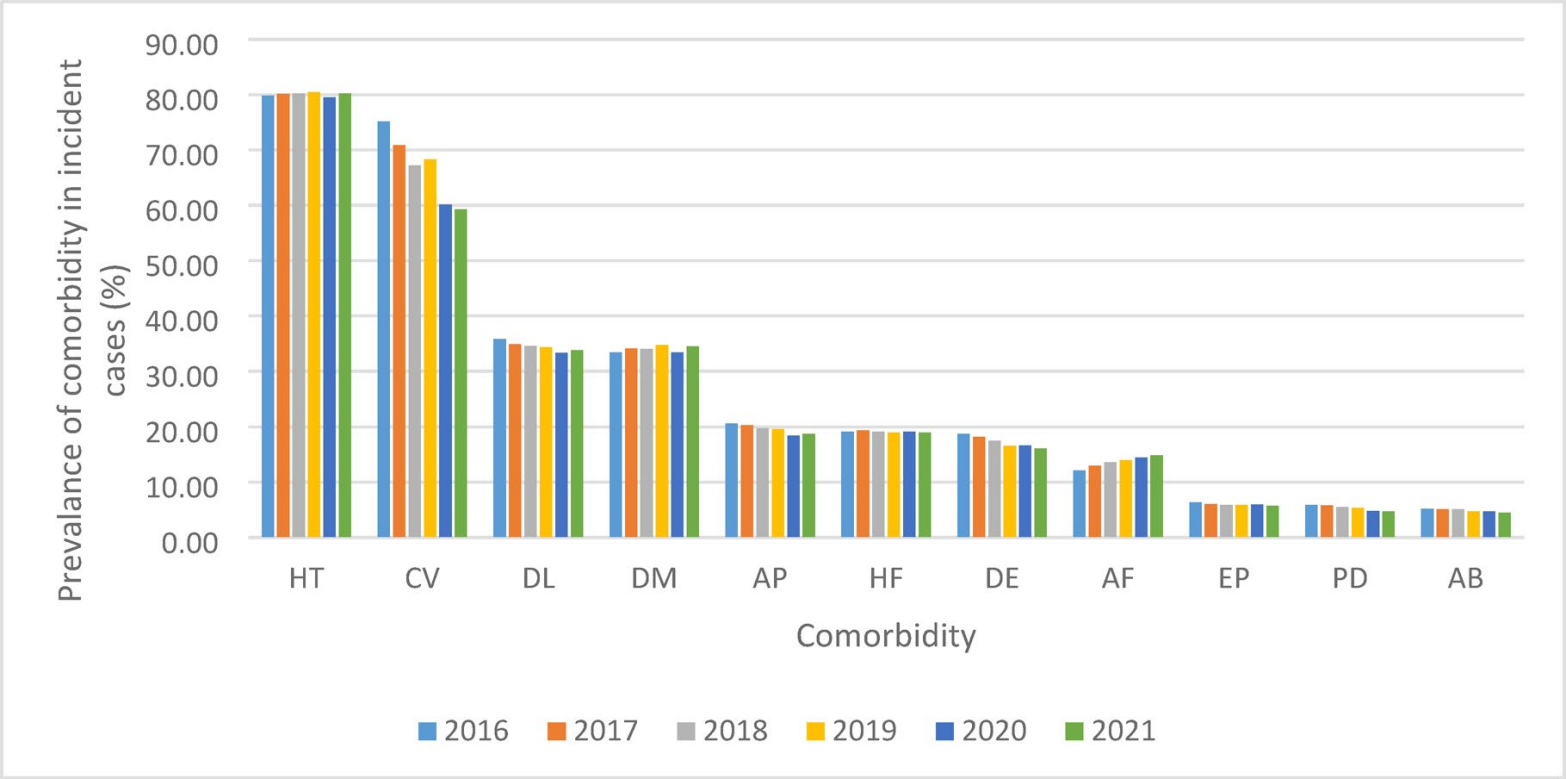
The prevalence of comorbidities within the population of newly diagnosed cases of NCD by year. *HT* Hypertension, *CV* Cerebrovascular diseases, *DL* Dyslipidaemia, *DM* Diabetes mellitus *AP* Angina pectoris, *HF* Heart failure, *DE* Depressive episode, *AF* Atrial fibrillation, *EP* Epilepsy, *PD* Parkinson’s disease, *AB* Mental and behavioural disorders caused by consumption of alcohol.

We investigated the prevalence of the most common comorbidities within the Hungarian NCD population (Fig. 4). The exact percentages for each year can be found in the Supplementary Table 3.

We found that in the various years, hypertension was diagnosed in 79.8%−80.5% of incident patients. No significant trend was observed. Cerebrovascular diseases co-occurred in 75.1% of patients in 2016, decreasing to 59.3% in 2021, showing a significant negative trend (APC − 4.65; *p* < 0.001,). The trend was also significant between the years of 2019 and 2021 (APC − 6.84; *p* < 0.001). Dyslipidaemia and diabetes were a comorbidity in one third (in a range of 33.3% − 35.8% and 33.4% − 34.7%, respectively) of newly diagnosed NCD patients, while angina pectoris and heart failure was present in one-fifth of NCD patients (in a range of 18.4% − 20.6% and 19.0% − 19.4%, respectively). Among these, only prevalent cases of angina pectoris showed a significant decreasing trend throughout the study period (APC=−2.19; *p* < 0.001). The prevalence of depressive episodes decreased from 18.7% to 16.1% with a significant negative trend (APC − 3.03; *p* < 0.001). The prevalence of atrial fibrillation and flutter increased from 12.1% to 14.83% with a significant positive trend (APC 4.00; *p* < 0.001). Epilepsy co-occurred in a range of 5.7% − 6.3% of incident NCD cases, showing no significant trend.

### Diagnostic procedures

**Fig. 5:**
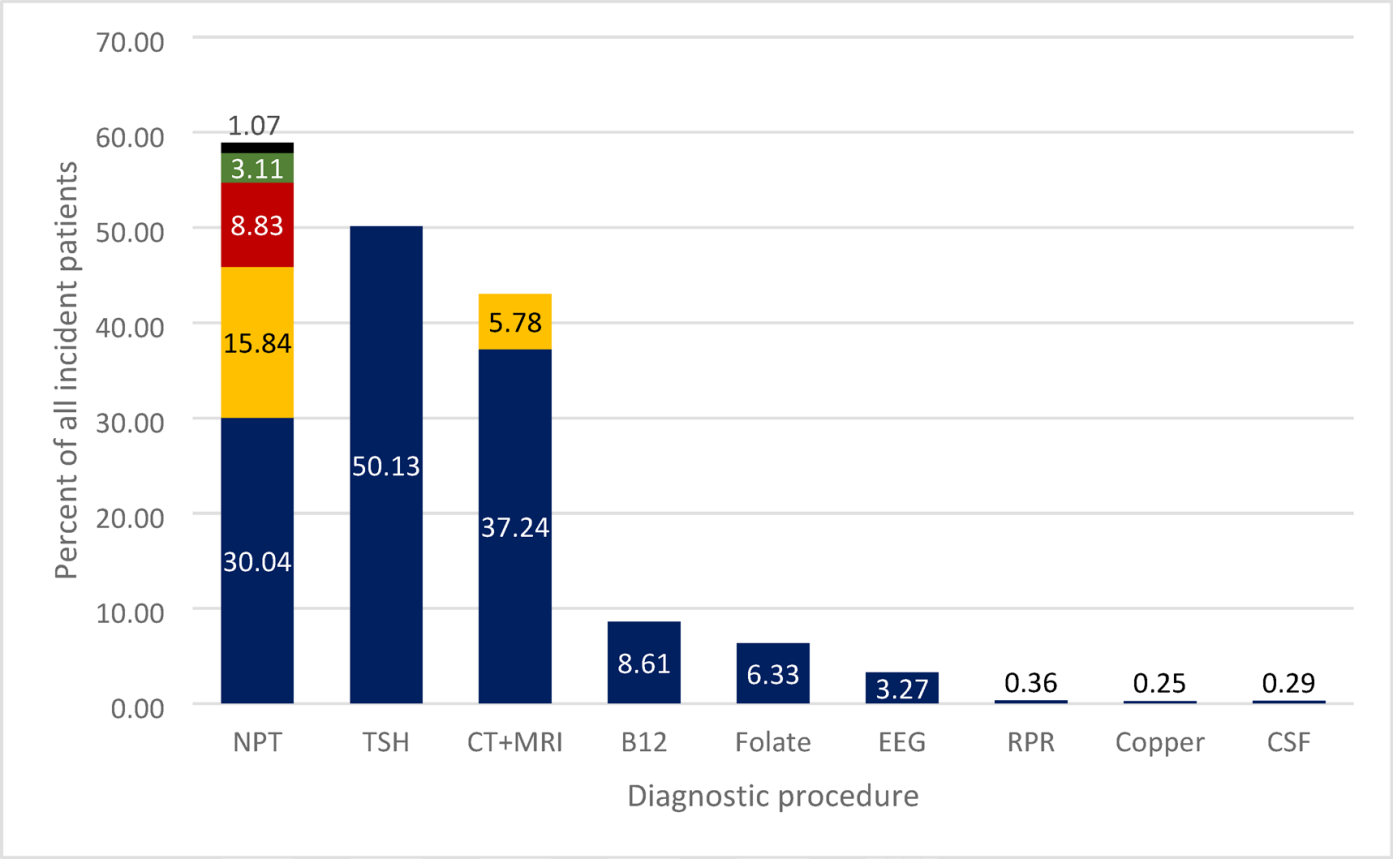
The proportion of all incident patients (2016–2021) who underwent a given diagnostic procedure in the year of or the year prior to diagnosis. Abbreviation: NPT: Neuropsychological tests- from top to bottom: General neuropsychological testing and problem-centred neuropsychological testing (summed), examination of dementia, mini-mental state exam, mapping of cognitive functions, psychiatric cross-sectional diagnosis; *TSH* TSH level in blood, *CT + MRI* Computer Tomography (bottom) and Magnetic Resonance Imaging (top), *B12* Cobalamin levels in the blood, *MRI* Magnetic Resonance Imaging, *Folate* Folic acid levels in blood, *EEG* Electroencephalography, *RPR* Rapid Plasma Reagin *Copper* Blood copper level, *CSF* Total protein in cerebrospinal fluid.

The data for diagnostic procedures performed on incident NCD patients over the study period (*n* = 312,781) is presented in Fig. 5. Data for each year can be seen in Supplementary Table 4. The most common diagnostic test in NCD patients was neuropsychological testing (NPT), which was performed in 58.89% of incident patients. Going from most to least common NPT test procedures: 30% was cross-sectional psychiatric diagnosis, 16% was mapping of cognitive functions, 9% was MMSE, 3% was the examination of dementia, and the remaining 1% was made up of problem-centred neuropsychological testing and general neuropsychological testing. The second most common procedure was the assessment of serum TSH level, which was carried out in 50.13% of incident patients. This was followed by CT and MRI imaging of the head, which was performed in 43.02% of incident patients (37.24% being CT and 5.78% being MRI investigation). The assessment of serum levels of vitamins of one-carbon metabolism was measured in 8.61% (vitamin B12) and 6.33% (folate) of incident patients. EEG was performed in 3.27%, and rapid plasma reagin test, serum copper level, and total protein content of the cerebrospinal fluid were performed in less than 1% of incident cases.

### Medication

**Fig. 6:**
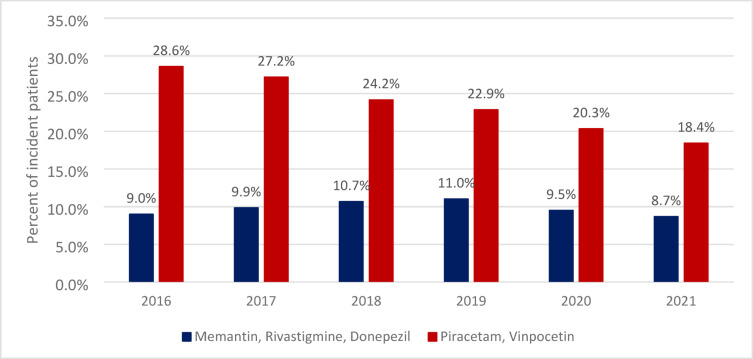
Percent of incident patients who filled at least 1 prescription of anti-dementia medication in the year of or the year following the diagnosis of NCD.

In incident cases of NCD, 9.0–11.0% (depending on the year) of patients filled their prescription of AChE inhibitor and/or NMDA receptor antagonist medication. This rate did not significantly change throughout the study period (Fig. 6). The proportion of patients who filled their vinpocetin and/or piracetam prescription in 2016 was 28.6%, which decreased to 18.42% in 2021 (significant negative trend: APC= −8.53; *p* < 0.001).

## Discussion

Our NCD prevalence results (~ 140,000–160,000) based on data received from the Hungarian National Health Insurance Fund were similar to the approximations for Hungary published in the Alzheimer Europe Yearbook 2019 (145,688)^3^. These results are in contrast with previous studies on the Hungarian population, where they estimated the Hungarian major NCD population to be 530,000–917,000^13,14^. This is most likely an overestimation since the data were obtained from a small number of general practitioners’ practices. Balázs et al. in 2021 found that the prevalence of NCD was 57,000 inhabitants^15^, however, their inclusion criteria were stricter, as they only considered cases where the patient had at least two diagnoses of NCD and at least one of these was done by a neurological or psychiatric specialty service. In contrast, our study included diagnoses made by any physician, and one diagnosis sufficed, thus, this explains the large difference in results between the two studies. In the Alzheimer Europe Yearbook 2019, they estimated the number of prevalent NCD cases to double by the year 2050^3^. Based on our results, this trend doesn’t seem to be holding, however considering our short study period and lack of raw AAPC (average annual percentage change) data it is difficult to predict with certainty. In our study 63% of NCD patients were women, which is consistent with previous studies^16^.

Based on our data, the prevalence of NCD cases decreased significantly after the start of the COVID-19 pandemic. The decrease in the number of diagnosed NCD patients after 2019 might be attributed to the coronavirus pandemic, much like in other European countries^15,17^, and may be partially explained by the lower rate of people seeking medical help during the pandemic^18^. This is despite the fact that the proportion of elderly people in the Hungarian population is continuously increasing: in 2016, 18% of the Hungarian population was 65 years of age and over, and by 2021, this increased to 21%^8^.

An even more prominent trend of decrease for incident NCD cases beginning at the latest in 2016 (well before the COVID-19 pandemic) was detected. People with major NCD suffering COVID-19 infection have over four times higher rate of death than people without major NCD^19^. This most probably contributes to the decrease in prevalence figures, however the total mortality for COVID-19 in Hungary was 48,762, which wouldn’t be high enough to explain the decrease in prevalence^20^. However, the reasons for the decline in incident cases in Hungary during the years preceding the pandemic remain unclear. No changes to the national diagnostic protocol for NCD have occurred since 2008; therefore, this trend cannot be attributed to modifications in diagnostic criteria or practice. In contrast, in other populations, such as the Flemish population, the number of incident cases increased prior to the pandemic^17^.

In Alzheimer Europe 2019, they estimated the number of Hungarian people to suffer from major NCD in 2018 under 65 years of age to be 9,318^3^. In our study in 2018, there were 42,811 prevalent cases of NCD. The difference can be partially attributed to the fact that our population included mild NCD cases as well, which are an antecedent stage of major NCD, and thus have an earlier age of onset than major NCD. Further explanation could be, that compared to other European population data, the Hungarian population has a much higher prevalence of comorbidities in incident NCD cases^21^. Our study also had a lower prevalence of NCD in the over 80 population than the estimations of Alzheimer Europe, which we believe can be attributed to underdiagnosis. Hypertension has a very high prevalence among incident cases of NCD, with 80% of the patients being affected. Cerebrovascular disease was found in 75% of patients in 2016, decreasing to 59% of incident patients in 2021 which might be partially explained by the decrease in the prevalence of stroke cases in Hungary from 43/100,000/year between 2005 and 2009^22^ to 31/100,000/year in 2023^21^. Diabetes and dyslipidemia were both found to be present in one-third of incident NCD cases. According to Browne et al.^23^, in the British population, the prevalence of these comorbidities in NCD patients was 53% for hypertension, 17% for stroke, and 14% for diabetes mellitus. All four of the aforementioned comorbidities are risk factors for NCD^24,25^, and their high occurrence rate in Hungary might contribute to the development of NCD in a larger portion of the population.

In a recent study, the ratio of vascular NCD to Alzheimer’s disease was estimated in the Hungarian population, and it was found to be 2.54:1^26^. This is surprising, taking into account that 2/3 of NCD cases are considered to be Alzheimer’s disease^27^. However, it can be explained by the high prevalence of vascular risk factors and cerebrovascular disease that we found in the Hungarian population.

Epilepsy was diagnosed in 5.7–6.3% of incident NCD patients, which is 8–11 times higher than in the elderly population without NCD (people over 65), where it is estimated to be 0.5–0.8%^28^. This supports previous data, which suggests that epilepsy is more than two to three times as common in patients with late-onset Alzheimer’s disease than in the general population^29,30^.

Even though neuropsychological evaluation is required for the diagnosis of NCD, these procedures were only performed in about half of the Hungarian NCD population. In the Hungarian protocol for diagnosing dementia, which was in effect during the period investigated in our study^10^, it is stated that a patient suspected to have NCD has to be tested at least by the MMSE. MMSE was performed in less than 10% of incident NCD patients. Furthermore, in order to exclude treatable aetiologies of NCD, the protocol also states that it is recommended to assess the serum level of TSH and vitamin B12, which was performed in 50.1% and 8.6% of the patients, respectively. The protocol also recommends that patients undergo structural imaging, preferably brain MRI. 43% of incident patients underwent structural imaging of the head, but only 6% of this was brain MRI. Since underdiagnosis in primary and specialist care, even in high-income countries like the UK, is about 45.5%^31^, and because of the low rate of crucial diagnostic procedures performed on patients before diagnosis, the actual prevalence of NCD in the Hungarian population may be different than what we observed in our study. The limited adherence to dementia diagnostic guidelines in Hungary is primarily driven by systemic and structural constraints rather than lack of clinical expertise. Time pressure, insufficient reimbursement, and restricted access to specialized diagnostic tools and neuropsychological services hinder comprehensive guideline-based assessments. Workforce shortages and regional disparities further limit implementation, particularly outside major centres. Consequently, clinicians often rely on simplified diagnostic approaches that deviate from ideal guideline recommendations.

Considering that the use of cholinesterase inhibitors postpones the institutionalization of AD patients by at least a year^32^ and that memantine use can save several thousand euros per year per AD patient in care costs (most likely by increasing quality-adjusted life-years and reducing the time spent in full-time care)^33^, adequate medication for individuals diagnosed with AD is essential. Our data suggest that in Hungary, only a small proportion of patients received approved anti-dementia treatment during the examined period (between 2016 and 2021), and many more used medications with disputed efficacy. Even if we consider that around 60–80% of major NCD cases are AD^34^, the proportion of patients receiving needed medication is still unexpectedly low. This might be explained by the fact that 65% of health care workers consider NCD to be a normal part of aging^35^, and thus may not consider it something to be treated. The number of patients taking vinpocetin and piracetam medication was steadily declining during the observational period. Since the decline started before 2019, the phenomenon is probably not related to the COVID pandemic. The 2008 Hungarian protocol for diagnosis and treatment of dementia^10^ allowed nootropic medication as a better-than-placebo option, while the new 2022 protocol^36^ states that such medication is not evidence-based in the treatment of AD. The change in the guideline and the observed decline in the prescribing of nootropic medications for NCD patients may reflect a change in overall attitude towards such medication among Hungarian physicians.

Our observations have significant implications for future health policy. For one, the high rate of comorbidities should guide policymakers to place elevated efforts on prevention to decrease the prevalence of NCD. Furthermore, the low proportion of diagnostic procedures for NCD can lead to the prescribing of superfluous medication for false-positive patients, and can also prompt the skewing of epidemiological data. The low proportion of patients filling their anti-dementia medication prescriptions should also be addressed by educating health and care workers about the importance of prevention and treatment of NCD-related diseases, so patients can receive adequate care.

There appears to be a lack of data on the epidemiology of NCD in the Central and Eastern European regions^4,37^, thus, our study might help to shed light on the state of affected populations in this region.

### Limitations

In light of our results about the low rate of diagnostic procedures performed on patients, who were diagnosed with NCD this calls into question how accurate the prevalence and incidence rates our that we acquired. Furthermore, since the data were obtained from the national healthcare system only people who reached a healthcare provider were diagnosed. In the UK this figure is about 45.5% of the NCD population^26^. In terms of medication, our data only reported on filled prescriptions of anti-dementia medication and does not describe prescription rates for NCD patients.

## Supplementary Information

Below is the link to the electronic supplementary material.

Supplementary Material 1

## Data Availability

The data that support the findings of this study are available from NEAK but restrictions apply to their availability. Data were used under license for the current study, and so are not publicly available. Data are however available from the authors upon reasonable request and with permission of NEAK.Data can be requested from: Christopher Váraljaiemail: christophervaraljai@gmail.com.
